# Outcomes in randomised controlled trials in prevention and management of carious lesions: a systematic review

**DOI:** 10.1186/s13063-017-2256-1

**Published:** 2017-11-02

**Authors:** Colin Levey, Nicola Innes, Falk Schwendicke, Thomas Lamont, Gerd Göstemeyer

**Affiliations:** 1 School of Dentistry, University of Dundee, Park Place, Dundee, UK; 2 Department of Operative and Preventive Dentistry, Charité-Universitätsmedizin, Berlin, Germany

**Keywords:** Systematic review, Caries, Core outcome set, Outcomes, Restorations, Caries prevention, Caries management, Carious lesions, Randomised controlled trial

## Abstract

**Background:**

Inconsistent outcome reporting is one significant hurdle to combining results from trials into systematic reviews. Core outcome sets (COS) can reduce this barrier. The aim of this review was to map outcomes reported in caries prevention and management randomised controlled trials (RCT) as a first step to COS development. We also investigated RCT characteristics and reporting of primary outcomes and sample size calculations.

**Methods:**

PubMed, Embase, Web of Knowledge and Cochrane CENTRAL were systematically searched (1 January 1968 to 25 August 2015). Inclusion criteria: RCTs comparing any technique for prevention or management of caries with another or placebo and RCTs comparing interventions to support patients undergoing treatment of caries (without setting, dentition or age restrictions). Categories were developed through piloting and group consensus and outcomes grouped accordingly.

**Results:**

Of 4773 search results, 764 were potentially relevant, full text was available for 731 papers and 605 publications met the inclusion criteria and were included. For all outcomes across the time periods 1968–1980 and 2001–2010, reporting of outcome ‘caries experience’ reduced from 39% to 18%; ‘clinical performance of the restoration’ reporting increased from 33% to 42% although there was a reduction to 22% in 2011–2015. Emerging outcome domains include ‘lesion activity’ and ‘pulp health-related outcomes’, accounting for 1% and 0%, respectively, during 1968–1980 and 10% and 4% for 2011–2015. Reporting ‘resource efficiency’ and ‘quality of life measures’ have remained at a low level. No publications reported tooth survival independent of an index such as DMFT or equivalent. Primary outcomes were only identified as such in 414 (68%) of the reports.

**Conclusions:**

Over the past 50 years, outcome reporting for trials on prevention and management of carious lesions have tended to focus on outcomes measuring caries experience and restoration material clinical performance with lesion activity and cost-effectiveness increasingly being reported. Patient-reported and patient-focused outcomes are becoming more common (although as secondary outcomes) but remain low in use. The challenge with developing a COS will be balancing commonly previously reported outcomes against those more relevant for the future.

**Trial registration:**

PROSPERO, CRD42015025310. Registered on 14 August 2015, Trials (Schwendicke et al., Trials 16:397, 2015) and COMET initiative online (COMET, 2017).

**Electronic supplementary material:**

The online version of this article (doi:10.1186/s13063-017-2256-1) contains supplementary material, which is available to authorized users.

## Background

Although preventable, dental caries continues to be one of the most common diseases globally, carrying substantial personal health impact and significant financial burden to treat [1–4]. Imperfections in the conduct and reporting of caries prevention and management trials has meant that systematic reviews have only been able to present poor or moderate quality evidence for even the most common preventive and management interventions [5–13]. Inadequate participants recruited (sample sizes), clinical heterogeneity, high risk of bias and inappropriate comparator or outcome choice within caries prevention and management trials have impacted negatively on the quality and strength of evidence [14]. Trials are conducted which can be combined together in systematic reviews to help create guidelines. It is therefore critical that action is taken to improve the quality of the evidence in the field of caries prevention and management. Different research groups, like Enhancing the QUAlity and Transparency of Health Research (EQUATOR) and Core Outcome Measures in Effectiveness Trials (COMET) have individually started to improve the quality of evidence creation and synthesis process. Overall, this approach follows a model of marginal gains [14].

One area where marginal gains can be made is in the harmonisation of outcome reporting in clinical trials. A trial outcome is the endpoint measured to allow comparison between the control and the intervention. It is what is measured (e.g. lesion activity), rather than how it is measured (the outcome measure, e.g surface texture index). Ensuring the best outcomes are chosen is central to the quality of the trial and the usefulness of the trial results.

A core outcome set (COS) is an agreed minimum group of outcomes which should be measured and reported for all trials involving a particular condition in a particular population. COS development began in medical fields in the 1990s [15] to address the problem of poor selection and reporting of trial outcomes. A Lancet Series on improving trial quality and reducing research waste [16–18] highlighted that ‘outcomes should be of importance to patients and not merely those that will show a statistically significant difference or are simplest to measure’. As well as harmonising outcomes to enable trial results synthesis in systematic reviews, a COS also reduces selective outcome reporting. This occurs when only statistically significant results are reported and statistically insignificant results are not. This can lead to a body of literature which overestimates treatment effect and under-reports harm [19–21]. This potentially skews the evidence and could have a significant, and negative, impact upon individual patients and healthcare systems, through the provision of costly, ineffective and/or perhaps harmful interventions.

In the dental literature, the importance of COS development is of growing interest [14, 22–25]. The first stage of COS development is to investigate outcomes that have previously been, and are currently, in use [26]. Therefore, there is a need to examine outcomes within the published literature around dental caries, look for trends and consider future priorities [27].

Stating and defining the primary outcome of a trial is a key recommendation of the CONSORT statement [28]. The primary outcome should be the outcome of greatest therapeutic importance [29]. It is used to calculate the number of participants required for the study to detect a difference between interventions and should be clearly identified a priori. Additional (secondary) outcomes may also be measured within a trial but are not the primary focus.

Prospective public trial registration is another recommendation of the CONSORT statement [28]. Trial protocols should be registered before commencing a study and can be used to confirm whether the study has been conducted as planned. In the context of outcome selection, the primary outcome in the report should be the one used in the registered protocol. This helps to identify outcome reporting bias.

The scope of this review is deliberately broad to capture a picture of outcomes to inform the COS development process. The primary aim of this review was to establish which outcomes have been used to assess treatment effect in randomised controlled trials (RCTs) of interventions for prevention or management of carious lesions in adults or children.

The secondary aims were:

1. to describe the characteristics of included trials; and
2. to assess compliance with reporting key aspects of the CONSORT statement related to handling of outcomes:
  - sample size calculations and their relationship with the primary outcome; and
  - trial registration.

## Methods

The protocol for this review was published in BMC Trials [26] and registered in PROSPERO (CRD42015025310) and on the COMET initiative website [30].

### Searches

Two search strategies were developed: the first focused on the prevention and the second on management of carious lesions (Table 1). Searches were developed and run individually for Embase, Web of Knowledge, PubMed and CENTRAL in August 2015 without language restrictions. Hand searching or assessment of the grey literature was not conducted for this review because it was considered unlikely to yield new outcome domains or significantly alter the overall results due to the high number of included publications.

**Table 1:** Medline search strategies

Prevention of carious lesions Medline search	
Search (((((((((((((fluoride) OR sealant) OR sealing) OR remineralisation) OR remineralization) OR remineralise) OR remineralize) OR antibacterial) OR chlorhexidine) OR brushing) OR brush))) AND (((((((((((((progression) OR prevention) OR arrest) OR prevent) OR progress) OR activity)))) AND (decay) OR carious) OR dmft) OR dmfs)))) AND ((((((patients) OR clinical) OR randomized) OR randomised) OR random)))))	
Management of carious lesions Medline search	
Search ((“Tooth”[Mesh]) AND “Dental Caries”[Mesh]) AND ((((((((((“pit and fissure sealant” OR “pit and fissure sealants”))) OR (“Pit and Fissure Sealants”[Mesh])) OR “Dental Restoration, Permanent”[Mesh]) OR “Dental Restoration, Temporary”[Mesh]) OR (((ultraconservative[Title/Abstract] OR stepwise excavation*[Title/Abstract] OR atraumatic*[Title/Abstract] OR minim*[Title/Abstract])) OR (ultraconservative[Text Word] OR stepwise excavation*[Text Word] OR atraumatic*[Text Word] OR minim*[Text Word]))) OR “Dental Cements”[Mesh]) OR “Dental Amalgam”[Mesh]) OR “Resins, Synthetic”[Mesh])	

### Inclusion criteria

We included RCTs involving any technique for preventing or managing carious lesions when compared to another intervention or no treatment. To capture patient-centred outcomes, we also included studies comparing interventions to support patients undergoing procedures related to caries. There were no restrictions on setting, time of follow-up, age or whether the study investigated the primary or permanent dentition. Artificial lesions and in situ studies were excluded. Screening of titles and abstracts was carried out independently and in duplicate by four authors (CL, FS, GG and NI) with agreement of two authors required for inclusion. Following screening for eligibility, 13 full text articles were not in English or German (languages spoken by the authors). As these made up only 1.7% of the total number of potentially eligible studies, they were excluded without translation.

### Data extraction

Data were extracted singly and independently by five authors (CL, FS, GG, NI and TL) following calibration using a pilot database. Independent extraction was chosen because numerical data were not being extracted and the risk of errors or missing outcomes was considered to be of low risk to data integrity. A 5% data check was undertaken and fields showing non-conformity > 10% were re-extracted after a further round of calibration. Due to the large number of included articles and the aim of our review, each article was included as a separate entity.

Data extracted:

- Trial details (author name, title, journal, date of publication);
- Trial characteristics;
  - ○ Study setting
  - ○ Number/age of participants
  - ○ Dentition
  - ○ Number of trial arms
  - ○ Interventions compared
  - ○ Prevention or management
- Outcomes assessed (collected as either stated primary outcome(s) or secondary outcome(s));
- Sample size calculation and whether it related to the primary outcome; and
- Trial registration.

Studies with interventions to avoid new lesions were assigned to prevention, while studies with interventions targeting existing lesions were assigned to management. An outcome was considered a primary outcome if it was stated as such or where the report clearly focused on one outcome. If no primary outcome was identifiable or multiple outcomes were reported these were considered secondary outcomes.

### Strategy for data synthesis

A list of outcomes was compiled and those with different verbatim terms but similar meanings were gathered using a single agreed term. Pilot category names were agreed before a first round of categorisation and refined through group consensus before all outcomes were re-categorised using the final agreed terms. The final list of outcome categories comprised 19 items and one ‘other’ category: caries experience (including DMFT, etc.); clinical performance of the restoration; lesion activity; microbiological outcomes; clinical oral hygiene-related outcomes (e.g. plaque and gingival indices); reaction to treatment during treatment procedure; pulp health-related outcomes; resource efficiency, e.g. time, cost; systemic side effects; pain/ discomfort, distinct from, and at a distance to, treatment; quality of life/subjective value; patient behaviour outcomes, e.g. toothbrushing frequency; patient knowledge; acceptability to operator; fluoride side-effects; aesthetics; service use; clinical diet-related, e.g. blood sugar levels; tooth survival; other.

Trial outcomes were allocated to one of these outcome categories by discussion and agreement of two authors (CL and NI). Where there was divergence, consensus was achieved through discussion with all five authors. A common area of difficulty was in deciding whether a particular outcome should be assigned to the caries experience or lesion activity outcome category. In such situations, if the outcome was related to caries progression within a single tooth or tooth level results, it was categorised as lesion activity. If it was reported at the patient level using a caries experience index, it was categorised as additional caries experience.

## Results

### Included studies

**Fig. 1:**
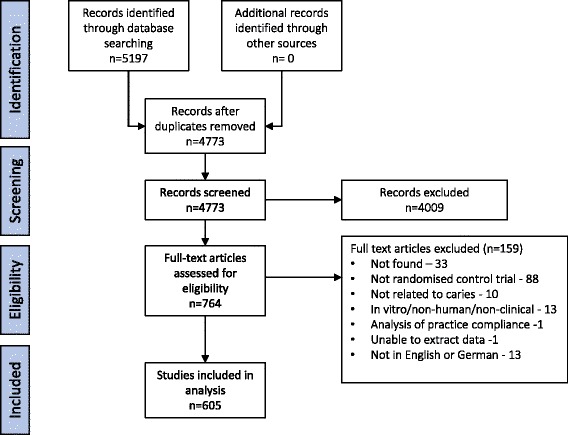
PRISMA *flow diagram* of search strategy and numbers of studies found, included and excluded (with reasons)

From the search, 5197 articles were identified across all databases and 4773 after duplicates removed. These were screened for eligibility independently and in duplicate by four authors (CL, FS, GG and NI) with agreement of two authors required for inclusion. There were 764 potentially relevant articles and the full texts of 731 were located (96% retrieval rate) with 126 of the retrieved articles excluded (not meeting inclusion criteria or not available in English or German). This left 605 full text articles in the final dataset for analysis (Fig. 1).

### Characteristics of included trials

#### Study setting

Of the 605 included reports, it was not possible to determine the study setting for 131 (22%) reports. Of the remaining 474, 73 (15%) were conducted in the primary care setting, 195 (41%) in secondary care (specialist practice, hospital or university), while 188 (40%) were set in a non-practice setting (field or school) and 18 (4%) in a mixed setting.

#### Number of participants

**Fig. 2:**
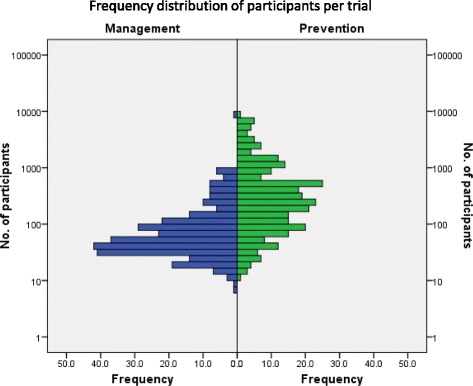
Logarithmic scale frequency distribution of the number of participants per trial (*n* = 252,099), by trial type. Participants in trials of caries management *n* = 43,282 (range = 7–8027) and for caries prevention trials *n* = 208,817 (range = 10–8027)

The total number of participants in the included trial reports was 252,099 (range = 7–8027 participants per trial), median 102 participants, mean 429 (interquartile range [IQR] = 284; qL44, qU328). Participant numbers were not normally distributed across the trials (Fig. 2). The total number of participants in prevention trial reports was 208,817 (range = 10–8027 participants per trial), median 257 participants, mean 735 (IQR = 512: qL99, qU611). The total number of participants in management trial reports was 43,282 (range = 7–8027 participants per trial), median 52 participants, mean 142 (IQR = 87: qL33, qU120).

#### Age of participants

Children (aged < 18 years) were the sole focus of 416 reports (69%) and adults in 154 (25%). Both adults and children were studied in 32 reports (5%). In three publications (0.5%) it was not possible to determine whether the participants were adults, children or both.

#### Primary/permanent dentition

Although 448 (74%) of the articles involved children (416 with children alone and 32 with both children and adults), the permanent dentition was studied in 447 (74%) of the trial reports (349 [58%] involving the permanent dentition alone and 98 [16%] for both the primary and permanent dentitions). The primary dentition was the focus in 150 (25%) of the studies. In eight (1%) of the reports, it was unclear which dentition was being studied.

#### Number of arms per trial

There were 415 (69%) reports describing trials with two arms; 124 trials (21%) had three arms, 44 (7%) had four arms and 22 (4%) of the reports had five or more trial arms.

More detail on the report characteristics of the included studies can be found in Additional file 1.

### Outcomes

#### All outcomes

**Fig. 3:**
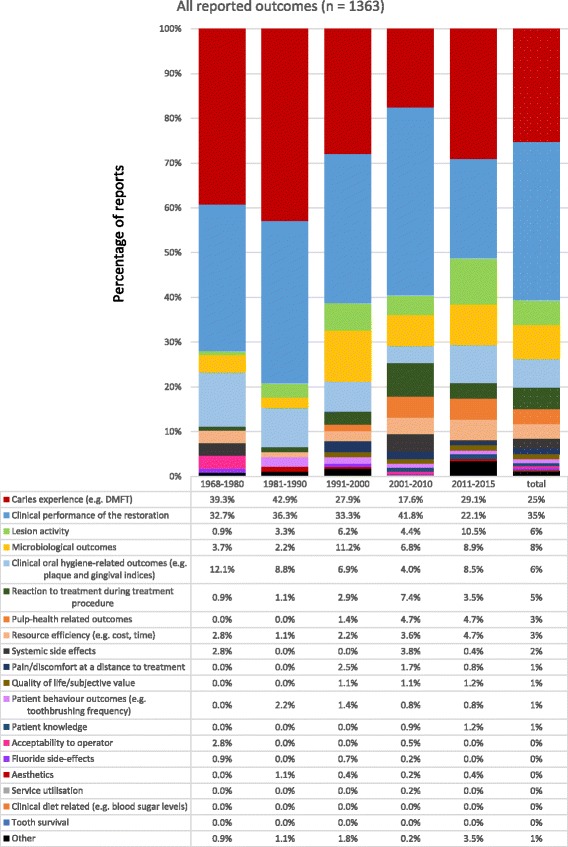
All reported outcomes (primary and secondary) for RCTs of carious lesion prevention and management (1968–2015), presented by domain and timeframes (*n* = 1364 outcomes)

There were 1363 outcomes reported in 605 published reports (Fig. 3). ‘Clinical performance of the restoration’ and ‘caries experience (e.g. DMFT)’ were the most common outcomes reported: 481 (35%) and 344 (25%) respectively. The next most common were ‘microbiological outcomes’ 103 (8%), ‘clinical oral-hygiene-related outcomes’ 87 (6%), ‘lesion activity’ 76 (6%) and ‘patient reaction to treatment during treatment procedure’ 66 (5%). There was around a 10% reduction each in the proportion of reports assessing ‘caries experience’ and ‘clinical performance of the restoration (from 1968–1980 to 2011–2015). At the height of their use (1981–1990), these outcome domains combined accounted for 79% of all reported outcomes, while most recently (2011–2015), this had fallen to 51%. Outcome domains that have increased in use include ‘lesion activity’, ‘microbiological outcomes’, participants’ ‘reaction to treatment during procedure’, ‘pulp health-related outcomes’ and ‘resource efficiency’.

### Primary outcomes

**Fig. 4:**
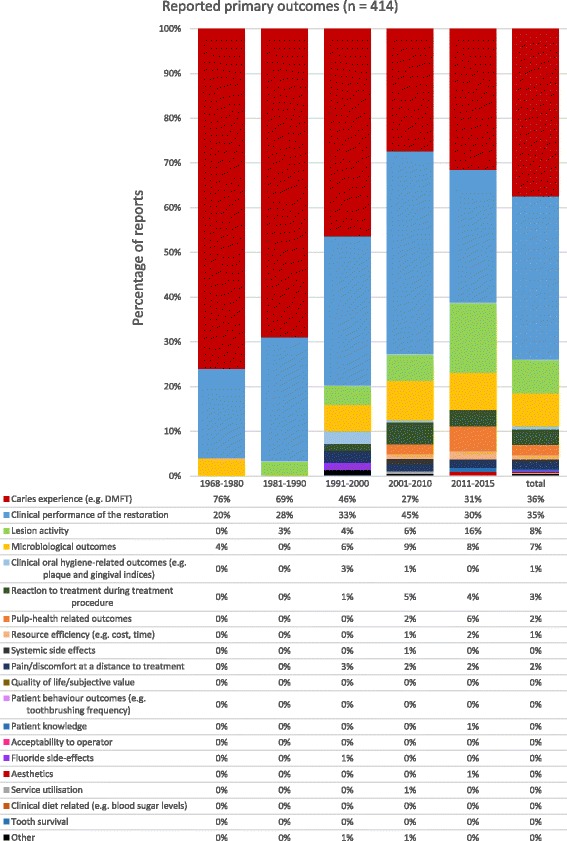
Primary outcomes reported in RCTs of carious lesion prevention and management (1968–2015), presented by domain and timeframes (*n* = 415 outcomes)

Primary outcomes could be identified in 414 (68%) reports (Fig. 4). Between 1968 and 2015, ‘clinical performance of the restoration’ was measured in 151 (35%) of these reports and ‘caries experience’ in 155 (36%), accounting for over two-thirds of the studies’ main focus. ‘Lesion activity’ was not measured in any studies in 1968–1980 rising from 3% (1981–1990), 4% (1991–2000), 6% (2001–2010) to 16% (2011–2015). Similar, although smaller increases are seen in ‘microbiological outcomes’, participants’ ‘reaction to treatment during treatment procedure’ and ‘pulp-health-related outcomes’, showing them also to be of growing interest as primary outcomes and the focus of the study.

### Sample size calculations and primary outcomes

**Fig. 5:**
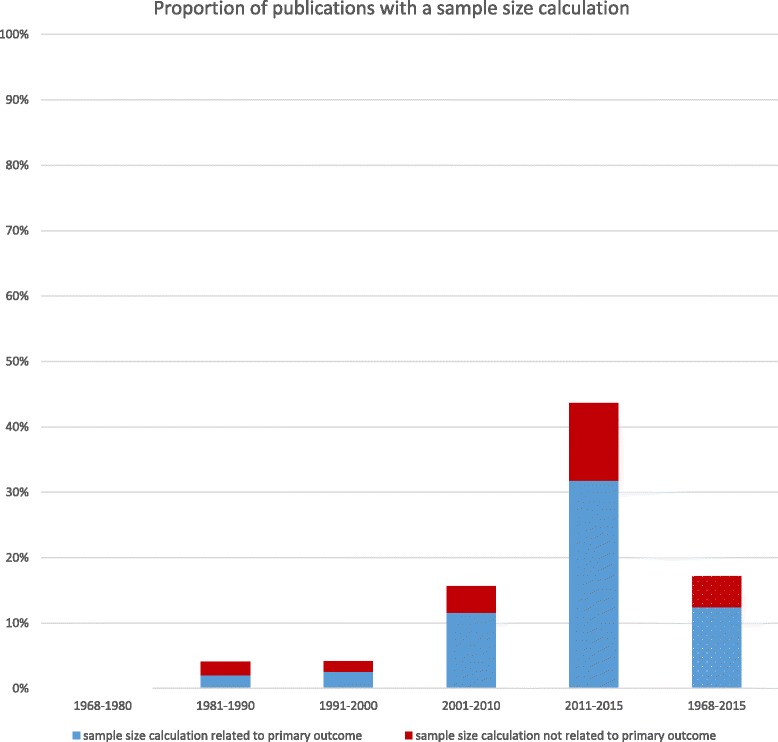
Percentage of publications with a sample size calculation for RCTs of carious lesion prevention and management (1968–2015) (*n* = 606 trial reports)

Information on sample size calculation was provided in 104 reports with the proportion increasing over time (Fig. 5). Fifty-five (44%) of all published reports in the 2011–2015 time period reported a sample size calculation. Overall (1968–2015) the sample size calculation was only linked to the primary outcome in 12% of all included reports.

Of the 104 reports which had a sample size calculation, 75 (72%) related to the primary outcome. The sample size calculation did not clearly relate to the primary outcome in 6% of publications and the information was unclear in 22% of reports.

### Trial registration reporting

Only 37 (6%) of all articles reported a trial registration in line with the CONSORT statement [28]. In the ten years following the publication of the first CONSORT statement in 2001, 3% of reports included a trial registration. Following the publication of the second CONSORT statement in 2010, this had risen to 21% (time period 2011–2015).

## Discussion

This systematic review of caries prevention and management trials’ outcomes gives a picture of RCTs over time. The outcomes that have been investigated show where emphasis has changed and which outcomes have been, and are becoming, of interest. It has also enabled identification of outcome domains for inclusion in a subsequent consensus process to determine a caries management COS.

The most commonly investigated outcome domains were ‘clinical performance of the restoration’ (assessed by the researcher or clinician) and ‘caries experience’ following an intervention. There was a paucity of trials where the outcome domains were patient-focused.

### Report characteristics

The vast majority of caries prevention and management interventions are provided in the primary dental care environment. However, only 15% of the reports (where this information could be determined) were conducted in this setting, with 41% set in secondary care. The represents an experimental focus on efficacy of interventions rather than effectiveness, within dental and oral healthcare. Non-practice settings such as schools provide a reliable participant group for caries prevention interventions involving large numbers of participants, which may explain why 40% of all reports were in this setting. This could also explain why most studies are carried out on only children and adolescents (69%) although the majority (74%) investigated the permanent dentition. The generalisability of studies carried out on young people’s permanent teeth (immature enamel, large pulp-to-crown ratios) to the vast majority of users of dental care services (adults) is questionable.

There was a wide range in the number of participants in each trial. The median was 102 and the mean 429 (IQR = 284). There were a relatively small number of studies with very large numbers of participants, skewing the data from a normal distribution. In general, prevention trials were larger than management trials with median participant numbers of 257 and 52, respectively. This may be because much larger sample sizes are required to show differences in preventively based interventions. They also tended to be population-relevant interventions such as school-based toothpaste/brushing programmes. The total number of participants involved in all of the trial reports was 252,099. This does not take into account multiple counting of participants because of trial reports being split across several publications and so is likely to be an overestimate. Although it is not possible to know how close this figure is to the actual number of participants, it is the most accurate figure we could achieve.

### Trial outcomes

Selecting the most appropriate outcome category in which to place each reported outcome presented a challenge which is consistent with other data in the oral health field [23]. Often, this was because the outcomes were poorly defined or were closely or inter-related [31]. Instances of overlap of outcome domains also resulted from composite outcome measurement tools. Indeed, this presents a potential challenge to COS development for carious lesion management, as the measurement tools for restoration performance (e.g. Ryge criteria) and further caries experience (Decayed Missing or Filled Teeth or Surfaces [DMFT or DMFS]) are frequently used as stand-alone outcomes rather than as a tool to measure a single clearly defined outcome. For example, DMFT tends to be used to describe ongoing caries experience, i.e. disease activity at the patient level, rather than simply tooth survival, although the latter is included in the ‘M’ assessment. It is perhaps surprising that tooth survival was not a standalone outcome in any study; that is, apart from its inclusion within a composite measure, e.g. DMFT. In this review, DMFT and similar indices were assigned to the caries experience category. Another common composite outcome measure, the Ryge criteria describes the clinical attributes and performance of restorative materials. This tool includes: a score for secondary caries development which could also be categorised as ‘caries experience’ or ‘lesion activity’; and marginal discolouration, which is an aesthetic outcome. We made consensus-led, clear decisions over how to categorise these outcomes into domains, aiming for clarity and consistency.

In some cases, the measurement tools appear to dictate the outcome domain and the outcome is poorly, or never, defined. A COS would harmonise the definition of outcome domains so that these can be compared in a meta-analysis. The COS development process should include clear definitions of outcomes to ensure consistency in application across future trials [32].

The outcomes reported in the caries literature from 1968 to 2015 have focussed on two areas; ‘clinical performance of the restoration’ (assessed by the researcher or clinician) and the amount of ‘caries experience’ following an intervention (often DMFT or similar). The importance of restoration performance is probably related to the rapid development of dental materials starting in the 1970s and continuing to the present day, whereas interest in measurement of amount of caries is likely to relates to fluoride toothpastes, gels and varnishes. Combined, these areas accounted for 74% of primary outcomes and for 61% of all reported outcomes. However, the use of these outcome domains appears to be reducing as a proportion of all reported outcomes with pulpal health-related and economic outcomes appearing to be of growing interest. These changes may reflect the development of, and interest in, minimally invasive approaches to lesion management and interest in cost/benefit balance in resource-limited healthcare settings.

Relatively few of the outcomes related to patient satisfaction or quality of life (1%), anxiety or pain during treatment (5%), discomfort after treatment (2%) or cost-effectiveness/resource use (3%). This shows that outcomes which are likely to be of importance to patients are not prominent in investigations. Encouragingly, patient-centred and patient-reported outcomes are increasingly being investigated and reported. This disparity strengthens the case for the development of a caries management COS which embraces the patient voice, as well as those from clinicians, researchers and other stakeholders.

Our review highlights the complexities in handling trial outcomes within the caries literature. For example, about one-third (32%) of reports did not have a discernible primary outcome so it was not possible to work out what the authors intended the main comparison of treatment success or failure to be. Even in the remaining 68%, the primary outcome was often not labelled as such, but was the focus of the report. Since the term ‘primary outcome’ has only relatively recently been adopted, we handled this by making a judgement on what the intended primary outcome seemed to be in these studies. This may not reflect an a priori primary outcome, but rather may be a result of an emphasis being placed, by the authors, on a significant result from an array of measured outcomes where the others were less interesting or not significant. If there was ambiguity over which outcome was the main outcome, all outcomes were classified as secondary. Therefore, the compliance with the CONSORT recommendations is likely to be even poorer than these results suggest.

A priori trial registration, a recommendation of the CONSORT statement [29, 33] would help with determining the intended primary outcome(s), reducing selective outcome reporting and improving standards within trials. Overall, only 6% of studies reported a trial registration and even taking a recent timeframe, between 2011 and 2015, only 21% of publications referred to trial registration.

Of 605 reports, only 104 gave a sample size calculation, and of these, only 75 related to the primary outcome. This means that almost nine out of ten studies (88%) did not report a sample size calculation at all or reported one that was not based on the primary outcome. Sample size calculations give a rational basis for the number of participants needed in the study to detect differences between the study interventions at an appropriate level of statistical confidence. They are a key recommendation in the CONSORT statement, published in 2001 [33] and revised in 2010 [29]. It is encouraging to note that, since its revision, 33% of publications had a sample size calculation which related to the primary outcome which is a noticeable change since pre-CONSORT statement levels (4%).

### Strengths and limitations of review

There is ongoing work investigating the most efficient systematic review methodology for outcome identification prior to COS development and it has been suggested that it may not be necessary to search multiple databases and that outcome saturation can be achieved by searching a subset of the literature [34]. However, in this review, we did search multiple databases and extracted data from all included trials. We did not carry out duplicate data extraction as the small gains in detecting errors in outcomes being recoded were not commensurate with the effort given our limited resources [35]. However, to reduce the impact of this we undertook a 5% data check.

It is standard practice for researchers to publish multiple reports based on data collected from the same clinical trial [36]. This can be necessary to report on the dataset at different timepoints but sometimes data collected for different outcomes are split across multiple publications. The complex task of linking registered protocols to specific reports is difficult and relies on aligning similar authorship and patient recruitment numbers. However, this becomes even more difficult when trials are not registered. We decided that while it was preferable to focus on each trial as a whole, rather than reports, this could not be done within our resources and was likely to have a limited impact on the integrity of the data.

There is no accepted caries outcome classification system and we derived the categories through an iterative process involving piloting the system then refining it until we agreed that the scheme was comprehensive but not too detailed and we had gained consensus on category names and allocation rules. Alternative classifications may have resulted in changes to the granularity and focus of the results. The broad scope of the categories may have resulted in unique outcomes being subsumed into larger categories. Any potential loss of information was mitigated during the consensus process when all authors were involved in suggesting novel outcome categories for consideration.

## Conclusions

Outcomes reported in RCTs for the prevention and management of carious lesions have tended to focus on the performance of restorative materials (e.g. Ryge criteria) or individual/population caries burden (e.g. DMFT or similar) related to an intervention. Outcomes related to carious lesion activity, pulpal health and economics have grown in use and are likely to be important in the future. Patient-reported/patient-centred outcomes are rarely reported although we found growing emphasis on outcomes of importance to patients. This deficiency reinforces the need for a COS with input from patients and other stakeholders during its development. The challenge with developing a caries lesion management COS will be balancing commonly reported outcomes (historic) against those more relevant for the future of dental care of the patient with dental caries.

## Additional files

Additional file 1: Included studies list and outcome catergorisation data. (XLSX 151 kb)

Additional file 2: Included study characteristics. (XLSX 78 kb)
